# A simple and efficient transient transformation for hybrid aspen (*Populus tremula* × *P. tremuloides*)

**DOI:** 10.1186/1746-4811-8-30

**Published:** 2012-08-07

**Authors:** Naoki Takata, Maria E Eriksson

**Affiliations:** 1 Umeå Plant Science Centre, Department of Plant Physiology, Umeå University, SE-901 87, Umeå, Sweden; 2 Present address: Forest Bio-Research Center, Forestry and Forest Products Research Institute, Hitachi, Ibaraki, 319-1301, Japan; 3 Department of Plant Sciences, University of Cambridge, Cambridge, CB2 3EA, UK

**Keywords:** *Populus*, *Agrobacterium*-mediated vacuum infiltration, Transient expression, Subcellular localization, Co-localization, Luciferase reporter assay

## Abstract

**Background:**

The genus *Populus* is accepted as a model system for molecular tree biology. To investigate gene functions in *Populus* spp. trees, generating stable transgenic lines is the common technique for functional genetic studies. However, a limited number of genes have been targeted due to the lengthy transgenic process. Transient transformation assays complementing stable transformation have significant advantages for rapid *in vivo* assessment of gene function. The aim of this study is to develop a simple and efficient transient transformation for hybrid aspen and to provide its potential applications for functional genomic approaches.

**Results:**

We developed an *in planta* transient transformation assay for young hybrid aspen cuttings using *Agrobacterium*-mediated vacuum infiltration. The transformation conditions such as the infiltration medium, the presence of a surfactant, the phase of bacterial growth and bacterial density were optimized to achieve a higher transformation efficiency in young aspen leaves. The *Agrobacterium* infiltration assay successfully transformed various cell types in leaf tissues. Intracellular localization of four aspen genes was confirmed in homologous *Populus* spp. using fusion constructs with the green fluorescent protein. Protein-protein interaction was detected in transiently co-transformed cells with bimolecular fluorescence complementation technique. *In vivo* promoter activity was monitored over a few days in aspen cuttings that were transformed with luciferase reporter gene driven by a circadian clock promoter.

**Conclusions:**

The *Agrobacterium* infiltration assay developed here is a simple and enhanced throughput method that requires minimum handling and short transgenic process. This method will facilitate functional analyses of *Populus* genes in a homologous plant system.

## Background

The genus *Populus*, which includes poplar, aspen, and cottonwood species, can serve as a model organism for perennial woody plants. *Populus* trees have several potential biological advantages such as small genome size, over 30 species growing worldwide, rapid juvenile growth, ease of clonal propagation, simplicity of genetic transformation and regeneration, and extensive genetic maps. The first draft genome of black cottonwood (*Populus trichocarpa*) was published in 2006 [1] and provided the first insights into the genomic organization of a tree species. The genome contains more than 45,000 protein-coding genes and ~12% of the genes show no similarity to genes in the model plant *Arabidopsis thaliana*[2]. To unravel the biological function of *Populus* genes, transgenic trees are generated and characterized, providing insight in unique characteristics, life style, and biological organization of perennial woody plants. However, a limited number of *Populus* genes have been targeted in transgenic studies due to a lengthy transformation process and the need for characterization of many transgenic lines for each construct.

Transient transformation assays complement stable transformation and make gene function analysis more efficient. Transient gene expression techniques (e.g., biolistic bombardment, protoplast transfection, and *Agrobacterium tumefaciens* (*Agrobacterium*)-infiltration) are available for model plants such as *Arabidopsis* and rice as well as for crop plants such as corn, potato, soybean, tomato, and wheat [3-7]. In *Populus* spp., several studies have successfully demonstrated transient gene expression via biolistic bombardment, protoplast transfection, and *Agrobacterium* co-cultivation [8-13]. For example, leaf epidermal and guard cells were transiently transformed with a reporter construct in a hybrid poplar (*Populus tremula* × *Populus alba*) by particle bombardment [12]. Transient transfection of *Populus* protoplasts isolated from leaf tissues and suspension culture cells was achieved either by means of electroporation or chemically with polyethylene glycol [8,10,12]. *Agrobacterium* co-cultivation was used to examine ectopic expression of a reporter gene in *P. nigraP. tomentosa*, and *P. trichocarpa* before the regeneration of stable transformants [9,11,13]. However, such transformation assays have certain disadvantages such as the requirement for expensive equipment and supplies associated with particle bombardment. In addition, the transformation efficiency is relatively low in the transfection of *Populus* protoplasts, and only a small piece of a tissue isolated from seedlings is used in *Agrobacterium* co-cultivation. To avoid these drawbacks, another transient assay such as *Agrobacterium*-mediated infiltration would be useful for *Populus* trees. This assay allows for a simple transformation process, easy operation, and high transformation efficiency in several plant species including *Arabidopsis*, grapevine, potato, switchgrass, tobacco, and tomato [6,7,14-17].

Transient transformation techniques are available for rapid *in vivo* analyses of gene function such as protein subcellular localization, protein-protein interaction, and promoter activity. For *in vivo* functional analyses, reporter genes such as green fluorescence protein (GFP), variants of GFP, and firefly luciferase (LUC) are common tools for molecular and cell biology studies. Protein subcellular localization, which is crucial for elucidating the cellular functions of proteins, is easily monitored by a transient expression of fluorescent fusion protein [18]. In this assay, a reporter construct harboring the gene of interest is fused with GFP or its variants and is transiently transformed into plant cells where intracellular localization is visualized through fluorescence of the reporter gene. Fluorescent proteins are also used for *in vivo* protein-protein interactions. Interaction assays, such as bimolecular fluorescence complementation (BiFC) and fluorescence resonance energy transfer (FRET), allow visualization of protein-protein interaction and subcellular localization of target proteins [19]. In these assays, transient co-transformation techniques with two different constructs are a convenient and practical alternative to generation of double-transformed transgenic plant and allow testing of several constructs and combinations. In addition to fluorescent proteins, LUC is used as a reporter gene mainly for measuring transcriptional activity. The LUC reporter is suitable for real-time monitoring of gene expression as it has a relatively short half-life compared to fluorescent proteins. For example, expression patterns of circadian clock-related genes, most of which show rhythmic expression in a day, are extensively examined by LUC reporter assays [20]. Typically, these assays use bioluminescence to visualize diurnal or circadian rhythms that express the LUC gene driven by a clock promoter of transgenic plants. Although studies regarding the plant clock system have principally been conducted using stable transformants, published studies report successful transient LUC assays [21,22].

In this study, we develop a transient transformation assay– *Agrobacterium*-mediated vacuum infiltration–for a model tree, hybrid aspen (*P. tremula* × *P. tremuloides*). The assay was optimized for aspen leaves and applicable to various cell types in leaf tissue. We investigated subcellular localization of *Populus* proteins and protein interaction by co-transformation of two different plasmids in aspen leaf cells. Furthermore, *in vivo* promoter activity of a hybrid aspen clock gene was measured using a LUC reporter assay. The transient transformation assay we developed is enhanced throughput and easily employed for rapid functional analyses of *Populus* gene and protein expression.

## Results and Discussion

### Transient transformation in hybrid aspen cuttings

We first examined whether the syringe injection technique, used in tobacco species, was useful for hybrid aspen leaves [14]. An infiltration medium [10 mM MgCl_2_ and 5 mM MES-KOH (pH 5.6)] in a blunt-tipped plastic syringe was forced into an abaxial epidermis of full-expanded aspen leaves that grew in soil for one month. The medium was permeated around the syringe contact area but limited to the wider area due to a leaf vein network (Additional file 1). Next, we investigated whether *Agrobacterium*-mediated vacuum infiltration increased the permeability of an infiltration medium for hybrid aspen leaves. Vacuum infiltration for transient gene expression has been systematic used in various plants such as *Arabidopsis*, citrus, lentil, switchgrass, and sunflower [15,17,23-25]. Briefly, hybrid aspen cuttings grown in the aseptic agar medium were submerged in an *Agrobacterium* solution with or without 0.003% Silwet L-77 and then vacuum-infiltrated. The infiltration solution penetrated throughout the aspen leaves by the aid of a low-pressure vacuum and the detergent (see below). Thus we could develop an *Agrobacterium* infiltration technique mediated by vacuum in aspen cuttings.

**Figure 1:**
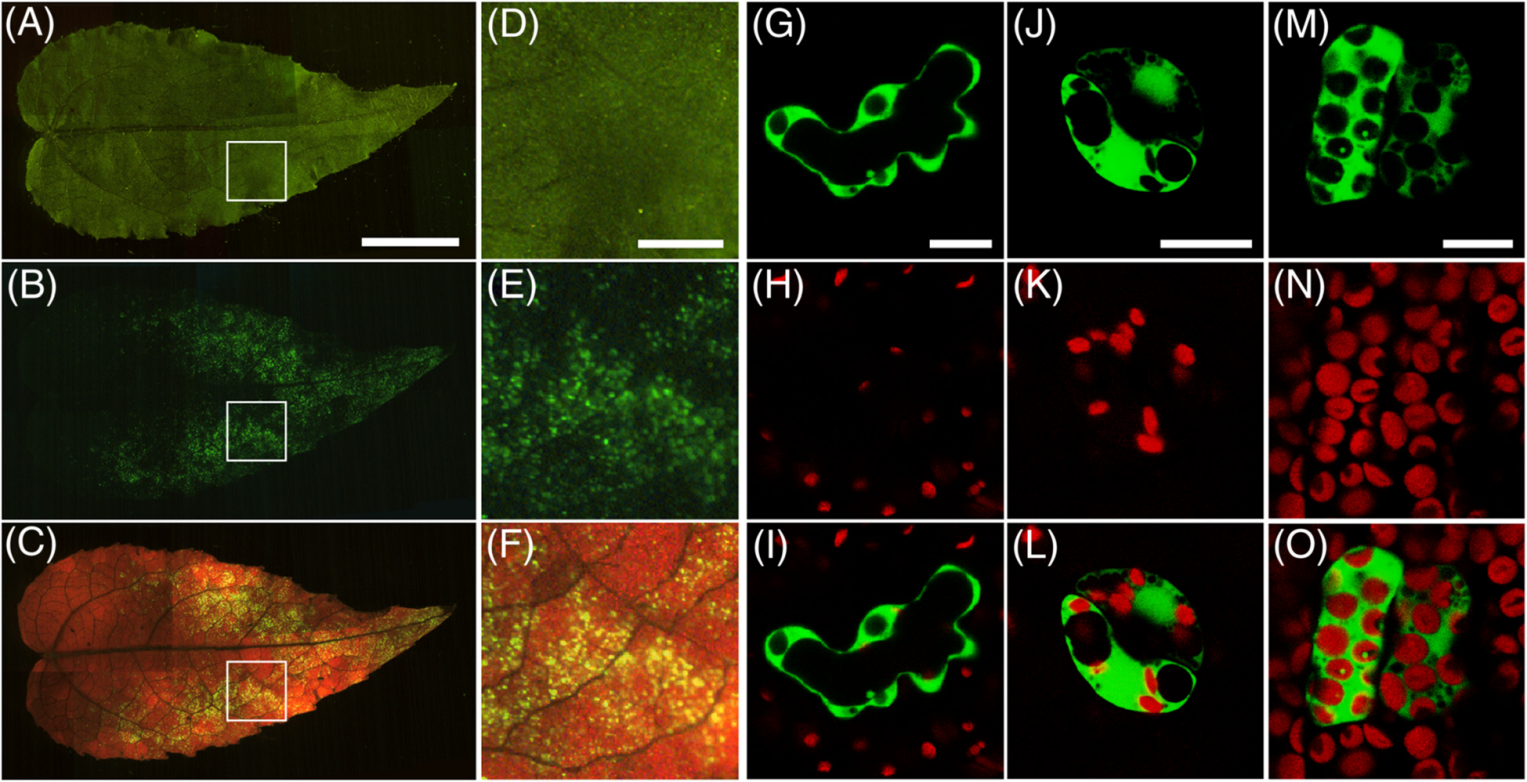
**Expression of GFP in hybrid aspen using *Agrobacterium* -mediated vacuum infiltration.(A-F)** Transient expression of GFP in aspen leaves. CaMV35S::EmGFP construct was transformed into hybrid aspen cuttings. Bright field image **(A** and **D)**, GFP fluorescence **(B** and **E)**, and GFP fluorescence and chloroplast autofluorescence **(C** and **F)** were captured using a fluorescence stereomicroscope. Scale bar = 5 mm **(A-C)**. Areas with a white box in **A**, **B**, and **C** are magnified in **D**, **E**, and **F**, respectively. Scale bar = 1 mm **(D-F)**. **(G-O)** Transient expression of GFP in various cell types in aspen leaves. GFP signals were observed in epidermal cells **(G-I)**, guard cells **(J-L)**, and mesophyll cells **(M-O)**. GFP fluorescence **(G**, **J**, and** M)** and autofluorescence of chloroplasts **(H**,** K**, and** N)** were captured using a CLSM. GFP signal and autofluorescence of chloroplasts were merged **(I**, **L**, and **O)**. Scale bar = 10 μm.

Transient transformation was monitored by the expression binary vector pPZP221-CaMV35S::EmGFP in the *A. tumefaciens* strain GV3101 (pMP90). *Agrobacterium* cells grown until stationary phase were re-suspended in an infiltration medium. Vacuum infiltration was performed using three- to four-week-old aspen cuttings without removing any tissues. When using a shoot detached from root tissues, the infiltration medium could not be evaporated from the intercellular spaces of the leaves and few cells were successfully transformed (data not shown). After three days of the transformation, GFP signals were principally observed in younger leaves and scattered in individual cells (Figure 1A-F). Most of the transformed cells were in the middle to tip region of the leaves. Although the intact plants were submerged and vacuum-infiltrated in the bacterial solution, few cells had GFP signals in petioles, stipules, stems, and roots (data not shown). GFP fluorescence was detected in various cell types such as epidermal cells, guard cells, and mesophyll cells in the leaf tissues (Figure 1G-O). This result indicates that the *Agrobacterium*-mediated vacuum infiltration technique can be used to analyze cells in young leaves. The validity of the tissue-dependent transformation is also described in *Agrobacterium*-mediated infiltration assay of *Arabidopsis*, where young cotyledons are more highly transformed than petioles and roots [15]. In the following studies, we principally used younger leaves for monitoring transformed cells.

### Conditions for efficient transient transformation in hybrid aspen leaves

**Figure 2:**
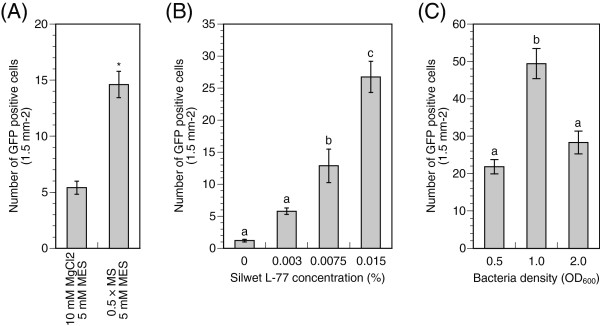
**Transient transformation efficiency in hybrid aspen leaves.(A)** Effect of infiltration medium on transient transformation. Overnight culture of *Agrobacterium* was re-suspended in individual infiltration medium containing 200 μM Acetosyringon and 0.0075% Silwet L-77 (final OD_600_ = 0.5). The GFP signals were observed after three days of the transformation. Transformation efficiency was calculated by counting GFP positive cells in a 1.5-mm^2^ leaf area. Values are means ± SE (n = 330). Asterisk indicates a statistically significant difference based on a Student’s *t*-test (P < 0.01). **(B)** Effect of Silwet L-77 concentration on transient transformation. Overnight culture of *Agrobacterium* was re-suspended in the medium [0.5 × MS, 5 mM MES-KOH (pH 5.6) and 200 μM Acetosyringon] (final OD_600_ = 0.5). Appropriate Silwet L-77 was added to the solution. Values are means ± SE (n = 350). Means with different letters are different according to Tukey’s HSD test (P < 0.05). **(C)** Effect of bacteria density on transient transformation. Overnight culture of *Agrobacterium* was re-suspended in the medium [0.5 × MS, 5 mM MES-KOH (pH 5.6), 200 μM Acetosyringon and 0.015% Silwet L-77]. The final OD_600_ was adjusted to 0.5, 1.0, and 2.0. Values are means ± SE (n = 480). Means with different letters are different according to Tukey’s HSD test (P < 0.05). The experiments shown are from two or three experiments each of which included three biological repeats. For each biological repeat, 40 to 60 sectors were measured.

We optimized the transformation conditions such as the infiltration medium, the concentration of Silwet L-77, the growth phase of bacteria, and the density of bacteria. Studies using other plant species report that these conditions influence the transient transformation efficiency in *Agrobacterium* infiltration technique [7,15,17,25,26]. We first estimated the effect of an infiltration medium on transformation efficiency. Two media were tested: (i) 10 mM MgCl_2_ and 5 mM MES-KOH (pH 5.6) established in an *Agrobacterium*-mediated vacuum infiltration of *Arabidopsis*[15] and (ii) 0.5 × MS medium and 5 mM MES-KOH (pH 5.6) based on the culture medium of hybrid aspen. A saturated overnight culture of *Agrobacterium* was re-suspended in the media containing 200 μM Acetosyringon and then diluted to a final OD_600_ of 0.5. Next, 0.0075% Silwet L-77 was added to the solution before applying vacuum. Three days after the vacuum-infiltration, GFP positive cells in the younger leaves were scanned and counted in small compartments (1.5-mm^2^ leaf area) to evaluate the transformation efficiency. The leaves transformed in the MS solution possessed 14.6 ± 1.2 (average ± SE) GFP positive cells per compartment (Figure 2A). However, fewer cells were transformed in the magnesium chloride solution (5.4 ± 0.6 cells per compartment). Hence, we decided to use the MS medium for further estimations of transient transformation.

Next, we investigated the effect of the surfactant Silwet L-77 concentration on the transient transformation efficiency. The presence of the surfactant allows a bacterial solution to penetrate into leaf lumen and boost the transient transformation efficiency, although excess surfactant accelerated necrosis of *Arabidopsis* leaf cells [26]. In this study, Silwet L-77 (0%, 0.003%, 0.0075%, 0.015%, and 0.03%) was added to the infiltration solution in which the bacteria were suspended (OD_600_ = 0.5) before the vacuum infiltration. As expected, few cells showed GFP fluorescence following transformation with media lacking Silwet L-77 because the penetration of the solution to the leaves was limited (Figure 2B). The Silwet L-77 concentration was positively correlated with the transformation efficiency and the highest number of transformed cells was obtained with 0.015% Silwet L-77 in the solution. However, in the medium containing 0.03% Silwet L-77, leaf cells appeared to be feeble and to be less transformed (data not shown). In the *Arabidopsis* transient transformation by co-cultivation with *Agrobacterium*, a similar positive correlation between the transformation efficiency and the Silwet L-77 concentration are reported and peak efficiency was established at 0.005% of *Agrobacterium* solution [26]. In this study, the surfactant concentration for aspen cuttings was three times higher than in *Arabidopsis*; this difference was probably because of different leaf structures and ages between species. In addition, transformation procedures were distinct from each other. We fixed the concentration at 0.015% of the *Agrobacterium* solution for aspen plants.

We determined the bacterial growth stage and density in the infiltration medium. The appropriate bacterial conditions lead to higher transformation rate in various plants, although the conditions depend on plant species [7,15,16,23,24,26]. To determine the appropriate bacterial growth stage, we harvested *Agrobacterium* at two growth phases–the log (OD_600_ = 0.8) and the stationary (OD_600_ = 1.6). The bacteria were re-suspended in the MS medium to an OD_600_ of 0.5. The leaf cells were successfully transformed in the stationary phase bacteria (data not shown). However, few transformed cells were obtained in the log phase bacteria, although an *Agrobacterium* at log phase growth was harvested and used to generate a stable transformant of hybrid aspen [27]. Next, we investigated the bacterial densities in the infiltration medium using the bacteria at the stationary phase growth. The final OD_600_ in the infiltration medium was adjusted to 0.5, 1.0, and 2.0. The highest transformation efficiency was achieved with a final bacterial density of OD_600_ = 1.0 (Figure 2v). When in the higher (OD_600_ = 2.0) and lower (OD_600_ = 0.5) bacterial concentration, the transformation yield dropped by half. Altogether, the simple and efficient transient transformation assay (the *Agrobacterium*-mediated vacuum infiltration) was optimized and developed for younger leaves of hybrid aspen, which were showing a higher incidence of transformation compared to older tissues.

### Subcellular localization of aspen proteins

One of the major applications of transient transformation assays is to observe the subcellular localization patterns of proteins in cells. Several studies have applied transient transformation to monitor subcellular localization of *Populus* proteins in heterologous plant systems [28-31]. However, few localization studies were tested in homologous *Populus* spp. Thus, we examined subcellular localization of *Populus* proteins in a homologous plant system using an *Agrobacterium*-mediated vacuum infiltration.

**Figure 3:**
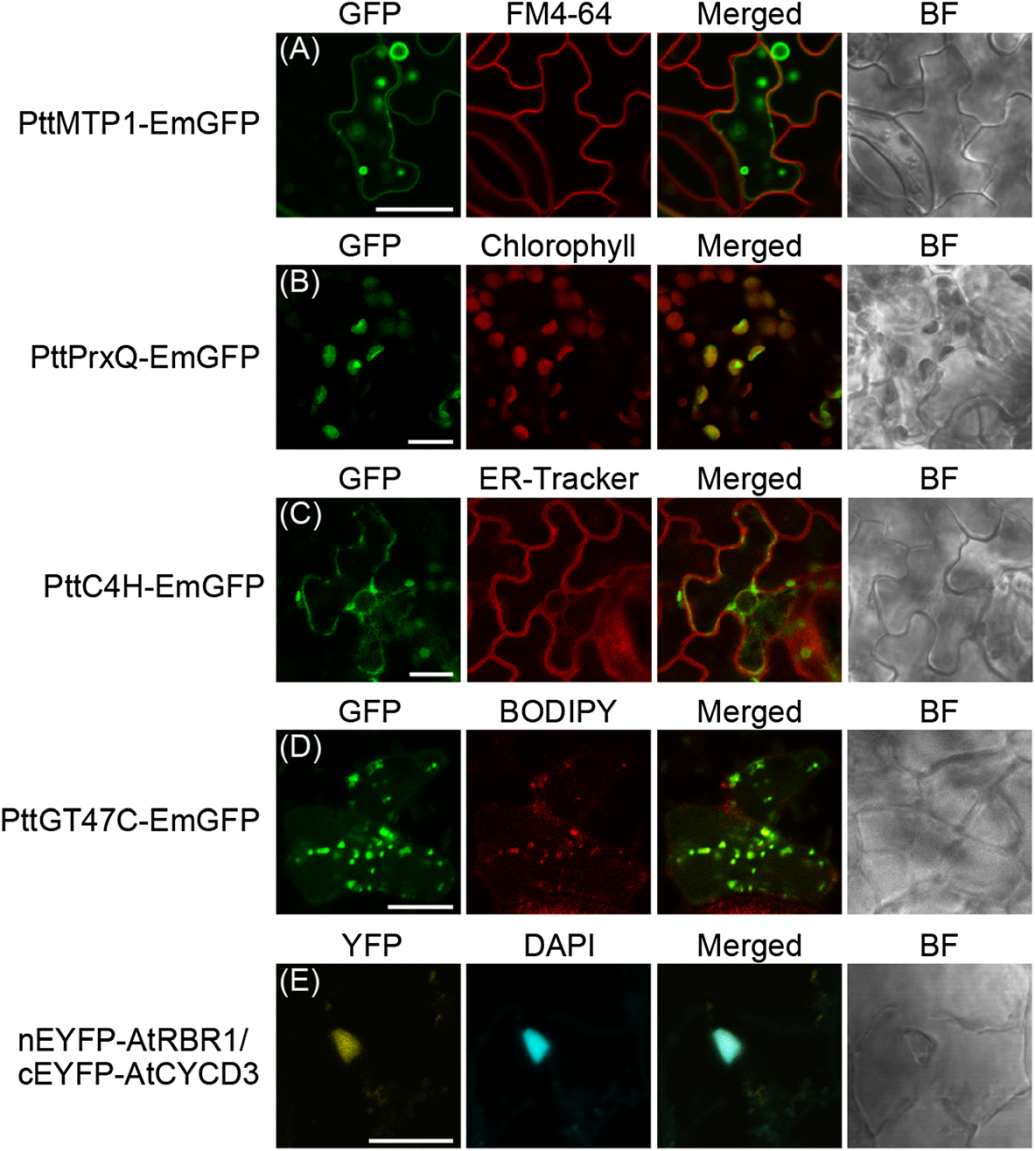
**Subcellular localization of *Populus* proteins and protein-protein interaction in hybrid aspen cells.(A-D)** Transient GFP fusion protein expression assay in hybrid aspen leaves. **(A)** PttMTP1-GFP labels the tonoplast, **(B)** PttPrxQ-GFP labels the plastids, **(C)** PttC4H-GFP labels the ER and **(D)** PttGT47C-GFP labels the Golgi. After three days of the transformation, GFP signals, chlorophyll autofluorescence and fluorescence of organelle markers were captured using a CLSM. Columns show GFP fluorescence (left), fluorescence of organelle markers and chloroplast autofluorescence (middle, left), the superimposed images (Merged; middle, right), and bright field images (BF, right). Scale bar = 10 μm. **(E)** BiFC assay in aspen leaves using transient co-transformation. The plasmid constructs containing CaMV35S::nEYFP-AtRBR1 and CaMV35S::cEYFP-AtCYCD3;1 were used for BiFC assay. YFP signals and DAPI fluorescence were captured using a CLSM. Columns show YFP fluorescence (left), DAPI (middle, left), the superimposed images (Merged; middle, right), and bright field images (BF, right). Scale bar = 10 μm.

This study examined the intracellular localization of four aspen genes–*CINNAMIC ACID 4-HYDROXYLASE* (*C4H*), *GLYCOSYLTRANSFERASE FAMILY 47* (*GT47C*), *METAL-TOLERANCE PROTEIN 1* (*MTP1*) and *PEROXIREDOXIN Q* (*PrxQ*)– which were previously described in heterologous plant expression system [28,29,32,33]. The expression vectors of C-terminal GFP tagged proteins were transiently transformed into hybrid aspen cuttings using *Agrobacterium*-mediated vacuum infiltration. We first determined the subcellular localization of the PttMTP1-EmGFP fusion proteins. The PttMTP1 protein, a vacuolar zinc transporter [28], was localized to the ring-like structure of the vacuolar membrane (Figure 3A). The tonoplast localization of the protein was distinguishable from the plasma membrane marked by lipophilic styryl dye FM4-64. The GFP signals of the PttPrxQ-EmGFP proteins overlapped with the autofluorescence of plastids (Figure 3B), in accordance with the finding that PrxQ is involved in detoxifying peroxides at the thylakoid membrane [29]. The PttC4H, a member of the cytochrome P450 monooxygenase superfamily, was distributed in the reticular network of ER labeled with ER Tracker (Figure 3C). The C4H protein targets ER in *Arabidopsis* and tobacco cells [32,34]. Finally, we tested the intracellular localization of the PttGT47C-EmGFP. The fluorescence from GFP-tagged PttGT47C exhibited a punctate pattern in the cytoplasm and was co-localized with the Golgi marker dye BODYPY TR ceramide (Figure 3D). In previous studies, *Arabidopsis* GT47C (FRA8) and poplar GT47C exhibited a similar localization pattern and served as a Golgi marker [33,35,36]. Therefore, the transient transformation protocol results showed reliability with respect to subcellular localization of the proteins selected to test targeting to various cell compartments.

### Co-transformation and protein-protein interaction by BiFC assay

We next investigated whether the transformation technique could be applicable to transient co-transformation assay in aspen leaves. To this end, we tested the BiFC assay, which is a powerful tool for visualizing protein-protein interactions in living cells. The transient BiFC assay is often performed via *Agrobacterium* infiltration technique in tobacco and *Arabidopsis*[15,26,37]. In this study, the validity of transient BiFC assay was evaluated using the *Arabidopsis* CYCLIN D3;1 (CYCD3;1) and RETINOBLASTOMA*-*RELATED 1 (RBR1) proteins for which an interaction has been shown in the nucleus [[38], Bakó László, personal communications]. A set of binary vectors for the BiFC assay–pGreen-CaMV35S::cEYFP-AtCYCD3;1 and pGreen-CaMV35S::nEYFP-AtRBR1–was transiently infiltrated into aspen cuttings. The EYFP fluorescence was detected in the nucleus co-localized with DAPI staining (Figure 3E), suggesting that AtCYCD3 and AtRBR1 formed a protein complex and functioned in the nucleus. However, co-transformation efficiency appeared to be lower than transformation with a single construct. This result suggests that two different constructs are successfully introduced into a single cell of hybrid aspen leaves and are viable for detecting protein interactions via BiFC using the *Agrobacterium*-vacuum infiltration technique.

### LUC reporter assay using *Populus* circadian clock gene promoter

Finally, we tested whether the transient transformation technique can be used in combination with the LUC reporter. We focused on rhythmic expression pattern of a circadian clock gene. Many circadian clock-related genes are identified in the model plant *Arabidopsis* and most of them show diurnal and circadian expression patterns in a phase-dependent manner [39]. The LUC reporter system easily and precisely estimates the rhythmic expression of a clock-related gene since gene expression can be followed as the LUC activity in the same plant over several days [20]. As the reporter system is generally applicable for a plant that stably expresses the *LUC* gene, a few studies have tested the possibility of the system using transient transformation assay [21,22].

**Figure 4:**
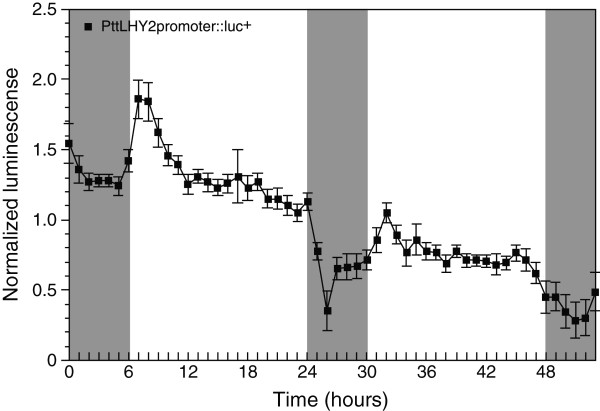
**LUC reporter assay in hybrid aspen apices.** The plasmid construct containing *PttLHY2*promoter::luc^+^ was transformed into aspen cuttings using the *Agrobacterium*-mediated vacuum infiltration assay. The apices were supplemented with luciferin solution at one day after the transformation. The LUC activity was detected by a cooled CCD camera beginning about 5 h following addition of substrate to obtain dynamic, real-time expression. Gray and white boxes indicate subjective night and day, respectively. Luminescence values are means ± SE (biological replicates n = 5).

We targeted the promoter of *Populus LATE ELOGATED HYPOCOTYL 2* (*LHY2*) gene, a key factor in the plant circadian clock system [40,41], in the transient LUC reporter assay. The reporter construct (*PttLHY2*promoter::luc^+^) was transformed into hybrid aspen seedlings by the *Agrobacterium*-mediated vacuum infiltration. The LUC activities were detected at one-hour intervals under light/dark cycles for two days. The *LUC* expression was robust in the shoot tips and the younger leaves (Additional file 2). In the leaves, the LUC activities in the young folded leaves were higher than those in the expanding and expanded leaves. The bioluminescence reflecting the *PttLHY2* gene expression showed a diurnal rhythm and a peak of the rhythm occurred 1–2 hours after dawn (Figure 4). The typical diurnal expression in the transformed aspen apices was followed over two days, although the luminescence level was reduced on the second day. A similar diurnal rhythm of *Populus LHY2* gene has been reported in the previous studies; these studies examined the expression level using the Real-time PCR technique [40,41]. Compared with the diurnal rhythm determined by Real-time PCR, more acute peaks in *LHY2* expression were monitored in the transient LUC assay, indicating that the assay would be a good tool for evaluating the phase and period of circadian expression. Taken together, the *Agrobacterium*-infiltration technique and the LUC reporter assay could be used to analyze driven, diurnal gene expression and could accelerate functional analysis of *Populus* clock-related genes.

## Conclusions

We have developed a novel transient transformation assay for hybrid aspen cuttings. For the *Populus* species, this simple assay has a higher throughput than the conventional transient assays [12]. Furthermore, this assay could be used to conduct quick functional analysis of protein subcellular localization, protein-protein interaction, and *in vivo* promoter activity. Although this technique is optimized in a wild-type plant, it can be applied to stable transgenic aspens, enabling the generation of a double mutant without additional long-term transformation process. Consequently, the transient transformation assay will facilitate functional analyses of *Populus* genes from various genera in a homologous plant system.

## Methods

### Plant material

Sterile rooted cuttings of hybrid aspen *P. tremula* × *P. tremuloides* (*Ptt*; wild type clone T89) were used for transient transformation assay. The cuttings were grown in 0.5 × Murashige and Skoog (MS) medium (pH 5.6) containing 0.8% (w/v) agar under a cycle of 18 h fluorescent light (50 μmol m-2 s-1)/6 h dark; 22°C/ 18°C.

### GFP-based constructs

The pUC18-based expression vector that harbors *EmGFP* gene (Invitrogen, USA) driven by the Cauliflower Mosaic Virus 35S (CaMV35S) promoter was provided by Dr. Tomokazu Yamazaki (Graduate School of Life Sciences, Tohoku University) [42,43]. The cloning sites (*Xba*I and *Bam*HI) between the CaMV35S promoter and *EmGFP* were altered into unique restriction sites (*Spe*I and *Kpn*I) by PCR-based site-directed mutagenesis [44] using the following primer set: forward, 5’-TACAGTCGACACTAGTGGTACCATGGTGAG-3’; and reverse, 5’-CTCACCATGGTACCACTAGTGTCGACTGTA-3’. To create a binary vector harboring CaMV35S::EmGFP, the pUC18-based vector was digested with *Eco*RI and *Hind*III and subsequently introduced into the *Eco*RI/*Hind*III site of the binary vector pPZP221 [45].

cDNA clones of *C4HGT47CMTP1* and *PrxQ* were isolated from *P. tremula* × *P. tremuloides* cDNA libraries [46]. Accession numbers of *PttC4HPttGT47CPttMTP1*, and *PttPrxQ* are BU883009, AI162472, BU893269, and BU813259, respectively. Coding sequence of these genes (without stop codon) were amplified by PCR using Platinum *pfx* DNA polymerase (Invitrogen, USA) with following primer sets: *PttC4H* (forward, 5'-CACCACTAGTATGGATCTCCTCCTCCTGG-3’; reverse, 5’-GGTACCAAAGGACCTTGGCTTTGCAAC-3’), *PttGT47C* (forward, 5'-CACCACTAGTATGAAACTATTACACAGACATGCT-3’; reverse, 5’-GGTACCGTGGTTTGAAAGCCTCACG-3’), *PttMTP1* (forward, 5'-CACCACTAGTATGGAAGCACAAAATCCTCAG-3’; reverse, 5’-GGTACCACGCTCTATCTGGATGGTTAC-3’) and *PttPrxQ* (forward, 5'-CACCACTAGTATGGCTTCCATTTCTCTCCC-3’; reverse, 5’-GGTACCAAGGCTTTGAAGTAGTTTAAGAGTT-3’). The PCR products were digested with *Spe*I and *Kpn*I and subsequently introduced into the *Spe*I/*Kpn*I site between the CaMV 35 S promoter and *EmGFP* of the expression binary vector pPZP221-CaMV35S::EmGFP.

### Agrobacterium infiltration

The expression binary vectors were transformed into *Agrobacterium tumefaciens* strain GV3101 (pMP90) [47]. *Agrobacterium* harboring individual vector was inoculated in LB media with appropriate antibiotics. An overnight culture of *Agrobacterium* was harvested at OD_600_ of 0.8 or 1.6, centrifuged at 4,000 × g for 20 minutes, and re-suspended in 25 mL of infiltration medium. This step was repeated. Finally, *Agrobacterium* cells were suspended in 100 mL of infiltration medium. Two infiltration media were used in this study: (i) 10 mM MgCl_2_, 5 mM MES-KOH (pH 5.6), and 200 μM Acetosyringon and (ii) 0.5 × MS medium, 5 mM MES-KOH (pH 5.6), and 200 μM Acetosyringon at OD_600_ of 0.5, 1.0, or 2.0. The bacterial solution was incubated at room temperature for three hours with gentle shaking under dark conditions. Hybrid aspen T89 cuttings (three to four week-old) were soaked in *Agrobacterium* solution containing 0%, 0.003%, 0.0075%, 0.015%, or 0.03% concentrations of Silwet L-77 (Lehle Seeds, USA) and then *Agrobacterium* infiltration was performed by applying vacuum (Boekel Scientific, USA) three times for three minutes. The aspen cuttings were subsequently put on paper towels to remove excess infiltration medium and transplanted in 0.5 × MS medium (pH 5.6) with 0.6% (w/v) agar and 50 μg/mL Cefotaxime. The cuttings were returned to grow under initial growing conditions for three days before imaging.

### Microscopy

Images of whole leaves were monitored by a fluorescence stereomicroscope (Leica MZ FLII, Germany) with a GFP3 filter (excitation 470/40, emission 525/50 nm) (Leica, Germany) for GFP fluorescence and a GFP2 filter (excitation 480/40 nm, emission 510 nm) (Leica, Germany) for GFP fluorescence and chloroplast autofluorescence. For the evaluation of transformation efficiency, GFP images in a 1.5-mm^2^ leaf area were captured using an epifluorescence microscopy (Axioplan 2 imaging, Carl Zeiss, Germany) with Zeiss filter set 09 (excitation 450–490 nm, emission 510 nm) (Carl Zeiss, Germany). GFP positive cells were counted in the small compartments using ImageJ 1.44 software [48].

### Staining and confocal microscopy

For confocal laser scanning microscopy (CLSM), GFP fluorescence, chloroplast autofluorescence and fluorescence of organelle markers in transformed cells were monitored using a Leica TCS SP2 AOBS system (Leica, Germany) with a water-corrected 63x objective NA = 1.2 (HCX PL APO 63.0x1.20 W BD UV, Leica, Germany) and a Leica TCS SPE system (Leica, Germany) with an oil-corrected 63x objective NA = 1.3 (ACS APO 63.0x1.30 OIL CS, Leica, Germany). For subcellular localization, transformed leaves were stained with plasma membrane marker FM4-64 (32 μM, Invitrogen, USA), ER marker ER-Tracker Blue-White DPX (1 μM, Invitrogen, USA) or Golgi marker BODIPY TR ceramide (5 μM, Invitrogen, USA). GFP and chlorophyll were excited at 488 nm laser. GFP and Chlorophyll emission were detected between 500 to 550 nm and between 664 to 696 nm, respectively. FM4-64 and BODIPY TR ceramide were excited at 532 nm laser. Fluorescence of FM4-64 and BODIPY dye were monitored between 640 to 660 nm and between 610 to 630 nm, respectively. ER-Tracker Blue-White DPX was excited at 405 nm laser and the emission was detected between 430 to 460 nm.

### BiFC assay

The BiFC vectors–pSAT1-nEYFP-C1-AtRBR1 (AGI code, At3g12280) and pSAT1-cEYFP-C1-B-AtCYCD3;1 (AGI code, At4g34160)–were provided by Dr. László Bakó (Umeå Plant Science Centre, Umeå University). The vectors were digested with *Nae*I and *Not*I and subsequently introduced into the *Eco*RV/*Not*I site of the binary vector pGreenI 0029 [49]. The binary vectors were transformed into *A. tumefaciens* strain GV3101 (pMP90). The *Agrobacterium* cells were inoculated in LB media with appropriate antibiotics and grown until the OD_600_ was above 1.8. The collected cells were washed twice by the MS infiltration medium and re-suspended in 50 mL of the medium at a final OD_600_ of 2.0. The same amount of the solutions were mixed and incubated at room temperature for three hours with gentle shaking. *Agrobacterium*-mediated vacuum infiltration was performed by the method described above. The transformed cuttings were grown on 0.5 × MS medium (pH 5.6) with 0.6% (w/v) agar and 50 μg/mL Cefotaxime for two days. To visualize nuclei, the leaves were stained with 4’,6-diamidino-2-phenylindole (DAPI, 5 μg/mL, Dojindo, Japan). EYFP fluorescence and DAPI fluorescence were detected using a Leica TCS SPE system with an oil-corrected 63x objective NA = 1.3. EYFP and DAPI were excited at 488 nm and 405 nm laser, respectively. EYFP and DAPI emission were detected between 510 to 550 nm and between 430 to 460 nm, respectively.

### Luciferase reporter assay

The promoter region of *P. tremula* × *P. tremuloides PttLHY2* was isolated by PCR with following primer sets: forward, 5'-TTAAGCTTTGCCTTCTGCAGATTTTCAG-3’; reverse, 5’-CGGGATCCTAGTGGACCTTAGGCAGCCA-3’) [Genbank/EMBL/DDBJ: AB661779]. The PCR products were digested with *Hind*III and *Bam*HI and subsequently introduced into the *Hind*III/*Bam*HI site of the binary vector pPZP221-luc^+^[50]. The binary vector was transformed into *A. tumefaciens* (GV3101 pMP90). *Agrobacterium*-mediated vacuum infiltration was performed by the same protocol described above. The transformed cuttings were grown on 0.5 × MS medium (pH 5.6) with 2% sucrose (w/v) and 0.8% (w/v) agar and brought back to standard growth conditions for a day following transformation. Subsequently, shoot tips with additional younger leaves were cut and transplanted on the same medium. The apices were supplemented with 5 mM luciferin in 0.01% Triton X-100 and placed under 18 h light (20 μmol m^-2^ s^-1^)/6 h dark; 22°C/ 22°C condition, light provided from red (660 nm) and blue (470 nm) light-emitting diodes (LEDs, MD Electronics, UK). Expression levels reflected by emitted photons were monitored using an ORCA-II-ERG 1024 cooled camera (Hamamatsu Photonics, Japan). Image acquisition and light control were driven by WASABI imaging software (Hamamatsu Photonics, Japan). The images were processed using Metamorph image-analysis software (Molecular Devices, USA).

### Statistical analysis

All statistical analyses were performed with R package version 2.9 [51].

## Abbreviations

BiFC, bimolecular fluorescence complementation; C4H, Cinnamic acid 4-hydroxylase; CaMV, Cauliflower mosaic virus; CLSM, Confocal laser scanning microscopy; CYCD3;1, CYCLIN D3;1; GFP, Green fluorescent protein; GT47C, Glycosyltransferase family 47; LHY2, Late elongated hypocotyl 2; LUC, Luciferase; MTP1, Metal-tolerance protein 1; PrxQ, Peroxiredoxin Q; RBR1, Retinoblastoma-related 1.

## Competing interests

The authors declare that they have no competing interests.

## Authors’ contributions

NT conceived of the study and drafted the manuscript. ME participated in planning, coordination and writing of the manuscript. Both authors read and approved the final manuscript.

## Supplementary Material

Additional file 1 Figure S1. Permeability of infiltration medium by syringe injection technique. The white circle indicates the syringe contact area. Click here for file

Additional file 2 Figure S2. The LUC activity in the shoot tip, folded and unfolded leaves. (A) Bright field image and (B) LUC bioluminescence eight hours after detection. Click here for file
