# Supplementary figures and images for: A simple and efficient transient transformation for hybrid aspen (Populus tremula × P. tremuloides)
Source: Plant Methods. 2012 Aug 7;8:30. doi: 10.1186/1746-4811-8-30 (PMC3476444; doi:10.1186/1746-4811-8-30)

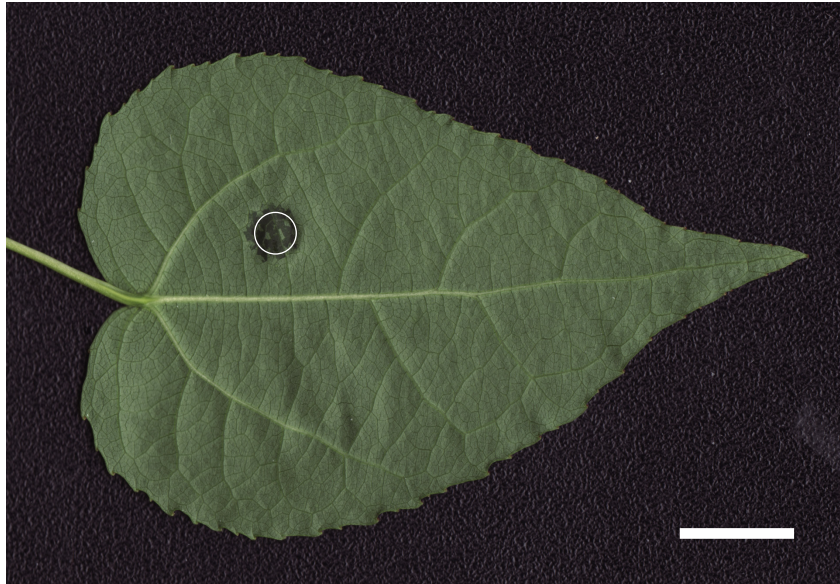

Bar = 1mm

Supporting Information Flg. S1. Takata and Eriksson

Supplement: Additional file 1 Figure S1. — Permeability of infiltration medium by syringe injection technique. The white circle indicates the syringe contact area. [file 1746-4811-8-30-S1.pdf]

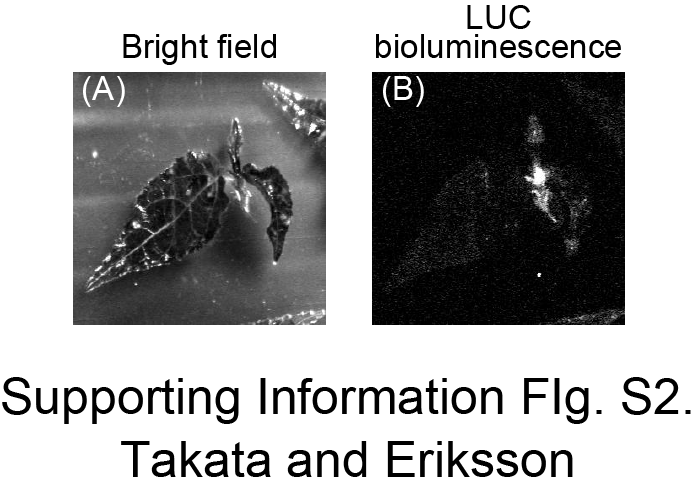

Supplement: Additional file 2 Figure S2. — The LUC activity in the shoot tip, folded and unfolded leaves. (A) Bright field image and (B) LUC bioluminescence eight hours after detection. [file 1746-4811-8-30-S2.TIFF]
